# Natural Hendra Virus Infection in Flying-Foxes - Tissue Tropism and Risk Factors

**DOI:** 10.1371/journal.pone.0128835

**Published:** 2015-06-10

**Authors:** Lauren K. Goldspink, Daniel W. Edson, Miranda E. Vidgen, John Bingham, Hume E. Field, Craig S. Smith

**Affiliations:** 1 Queensland Centre for Emerging Infectious Diseases, Biosecurity Queensland, Department of Agriculture and Fisheries, Coopers Plains, Queensland, Australia; 2 Australian Animal Health Laboratory, Commonwealth Scientific and Industrial Research Organisation, East Geelong, Victoria, Australia; 3 EcoHealth Alliance, New York, New York, United States of America; Metabiota, UNITED STATES

## Abstract

Hendra virus (HeV) is a lethal zoonotic agent that emerged in 1994 in Australia. Pteropid bats (flying-foxes) are the natural reservoir. To date, HeV has spilled over from flying-foxes to horses on 51 known occasions, and from infected horses to close-contact humans on seven occasions. We undertook screening of archived bat tissues for HeV by reverse transcription quantitative polymerase chain reaction (RT-qPCR). Tissues were tested from 310 bats including 295 Pteropodiformes and 15 Vespertilioniformes. HeV was detected in 20 individual flying-foxes (6.4%) from various tissues including spleen, kidney, liver, lung, placenta and blood components. Detection was significantly higher in *Pteropus Alecto* and *P*. *conspicillatus*, identifying species as a risk factor for infection. Further, our findings indicate that HeV has a predilection for the spleen, suggesting this organ plays an important role in HeV infection. The lack of detections in the foetal tissues of HeV-positive females suggests that vertical transmission is not a regular mode of transmission in naturally infected flying-foxes, and that placental and foetal tissues are not a major source of infection for horses. A better understanding of HeV tissue tropism will strengthen management of the risk of spillover from flying-foxes to horses and ultimately humans.

## Introduction

Hendra virus (HeV) is a paramyxovirus of the genus *Henipavirus* responsible for fatal infection in horses and humans in eastern Australia. Flying-foxes of the genus *Pteropus* have been identified as the reservoir host of HeV through serological surveys [1–3] and the isolation of virus in naturally infected *Pteropus poliocephalus* and *P*. *alecto* [2]. HeV antibodies have been identified in all four Australian mainland *Pteropus* species; *P*. *alecto*, *P*. *poliocephalus*, *P*. *scapulatus*, and *P*. *conspicillatus*. No evidence of HeV infection has been found in non-pteropid bats [1,2,4]. Since its discovery in 1994, HeV has spilled from its natural reservoir host on 51 known occasions to 30 June 2014 [5] resulting in predominantly respiratory and neurological disease (typically fatal) in horses [6]. In a minority of cases, infected horses have transmitted HeV to close-contact humans, causing similar disease profiles with a case fatality rate of 57% [7,8]. There is no evidence that HeV causes clinical disease in either naturally infected [4] or experimentally infected flying-foxes [9–11].

Despite significant research efforts over the past 20 years, the exact mode of HeV transmission between flying-foxes, or from flying-foxes to horses, has not been conclusively determined. Several studies have investigated potential routes of excretion, tissue tropism and transmission of infection in both experimentally and naturally infected flying-foxes [2,9–11]. HeV has been identified in the lung, spleen, liver, and kidney of experimentally infected adult *P*. *alecto* [11] and in the kidney, spleen, heart, and vascular tissue of experimentally infected *P*. *poliocephalus* [9,10]. HeV has also been detected in naturally infected flying-foxes [12] including the uterine fluid and pooled foetal lung and liver from aborted twin foetuses of one *P*. *poliocephalus*, and the foetal lung of one *P*. *alecto* [2]. There are a number of biologically plausible modes of HeV excretion in flying-foxes, including infected placental tissue, placental fluids, urine, faeces and oronasal secretions. The predominant hypotheses regarding HeV spillovers involve equine exposure to 1) infected placental and birthing material, or 2) infected excreta (i.e. urine, faeces, spats) or partially-eaten food.

The majority of studies that examine risk factors for spillover events have used serological data or modelling to assess flying-fox infection dynamics over time. Serological surveys of wild *P*. *conspicillatus* [13] and *P*. *scapulatus* [14] populations have shown increased detection of HeV antibodies associated with late-stage gestation and/or early lactation. Consequently, gestation and birthing seasons in flying-foxes have been broadly accepted as risk factors for increased HeV prevalence, and for spillover risk. Although these studies related to *P*. *scapulatus* and *P*. *conspicillatus*, this assumption has been extrapolated in risk modelling involving *P*. *alecto* and *P*. *poliocephalus* [15].

Understanding how natural infection may differ from experimental models, and identifying potential risk factors for HeV infection in its natural host is fundamental to accurately assessing the risk of spillover from flying-foxes to horses. While there has been a tendency for all flying-foxes to be considered equal in terms of their role as the natural HeV host [15], recent spatial modelling of spillover events indicates that the presence of only *P*. *alecto* and *P*. *conspicillatus* correlate with spillovers [16].

There remain important gaps in our knowledge of HeV infection dynamics at both the individual flying-fox level and the population level, and how they influence the risk of spillover to horses. To date our knowledge of individual flying-fox infection dynamics of HeV is derived from experimental and, to a lesser extent, natural infections from a limited number of species and animals. Population-level infection studies of HeV in flying-foxes primarily utilise seroprevalence as a surrogate for infection. How experimental infection compares with natural infection within the four *Pteropus* species remains largely unknown.

This study screens an archived collection of bat tissues for molecular evidence of natural HeV infection and analyses the data to determine tissue tropism and risk factors associated with its detection in flying-foxes.

## Materials and Methods

### Sample Collection

Homogenised tissue samples were obtained from an archive of 310 bats, comprising both Pteropodiformes (n = 295) and Vespertilioniformes (n = 15), collected in Queensland between 1996 and 1997 (Table 1) under (then) Department of Primary Industries and University of Queensland animal ethics permits [1,2]. No animals were killed specifically for this study. The collection included brain, kidney, liver, lung, spleen, placental and selected foetal tissues; red blood cells (packed haemocytes including the buffy coat fraction) and serum; oral and prepucial swabs; uterine scrapings; and urine samples. All tissues had previously tested negative for virus by cell culture and for viral RNA by quantitative RT-PCR [2]. All tissue extraction work was carried out in a NATA-accredited BSL3 facility, in accordance with AS/NZS 2243.3:2010. These tissues were held on liquid nitrogen in long-term storage. Some tissues had duplicate formalin fixed tissues embedded in paraffin wax blocks.

**Table 1:** Test results from RT-qPCR (n = 310) were subjected to a generalised linear model [19] under the Binomial distribution and logit link, using GenStat [20].

Variable	Category	Detected(Total)	Adjusted mean prevalence (±S.E.)	P χ^2^
Reproductive status				0.122
	Male	6 (119)	4.7 (2.9–6.5)	
	Immature female	0 (36)	0.0 (0.0–0.1)	
	Mature female not pregnant	8 (72)	9.4 (6.3–12.5)	
	Mature female pregnant	4 (41)	10.8 (6.1–15.5)	
	Unknown	2 (40)	6.2 (2.2–10.3)	
Season				0.689
	Spring	7 (146)	7.1 (4.5–9.7)	
	Summer	6 (74)	4.7 (2.7–6.6)	
	Autumn	2 (41)	12.2 (5.2–19.1)	
	Winter	5 (49)	7.1 (4.1–10.1)	
Species				0.003
	*Chalinobus spp*.^a^	0 (2)	0.0 (0.0–0.2)	
	*Miniopterus australis* ^a^	0 (1)	0.0 (0.0–0.3)	
	*Nyctophilus spp*.^a^	0 (1)	0.0 (0.0–0.3)	
	*Pteropus alecto* ^b^	9 (105)	8.6 (5.8–11.3)	
	*Pteropus conspicillatus* ^b^	10 (44)	22.7 (16.4–29.0)	
	*Pteropus poliocephalus* ^b^	1 (91)	1.1 (0.0–2.2)	
	*Pteropus scapulatus* ^b^	0 (50)	0.0 (0.0–0.1)	
	*Saccolaimus flaviventris* ^a^	0 (1)	0.0 (0.0–0.3)	
	*Scotorepens spp*.^a^	0 (3)	0.0 (0.0–0.2)	
	*Synconycterus spp*.^a^	0 (3)	0.0 (0.0–0.2)	
	Unknown *Pteropus* spp.^b^	0 (5)	0.0 (0.0–0.1)	
	Unknown Vespertilioniformes^a^	0 (4)	0.0 (0.0–0.2)	
Latitude				0.232
	North	13 (82)	9.3 (5.9–12.7)	
	South	7 (228)	4.2 (2.4–6.0)	
Year				0.233
	96	7 (105)	4.7 (3.0–6.4)	
	97	13 (205)	8.0 (6.0–9.9)	

Adjusted mean proportions, and their standard errors are reported.

^a^Vespertilioniformes.

^b^Pteropodiformes.

### Tissue Screening

Based on previous demonstration of urine as an efficient sample for HeV surveillance [12], kidney tissue was used for initial screening. All animals with HeV-positive results on kidney had their full complement of tissues tested. Due to extensive detections in spleen tissue in this phase, all 310 animals were retested using spleen as a second screening tissue. Kidney and spleen samples from all animals, including foetal kidney and spleens, were tested for HeV as described below. Animals where HeV was not detected in kidney or spleen did not have their remaining tissues tested, however all available placental tissues were tested.

### RNA Extraction

The MagMax-96 Viral RNA Isolation Kit (Life Technologies) was used on a magnetic particle handling system (Kingfisher 96) to extract total nucleic acid from tissue homogenates (50μL) as per the manufacturer’s instructions. The purified nucleic acids were eluted in 50μL of elution buffer and used immediately for PCR amplification.

### Reverse Transcription Quantitative Polymerase Chain Reaction

The AgPath-ID One-Step RT-qPCR Kit (Life Technologies) was used for RT-qPCR to a total volume of 25μL. Forward and reverse primers and probe that target a 69 base pair region on the M gene were used as described by Smith *et al*. (2001) [17]. Positive and negative controls were included for quality assurance. RNA (5μl) was added to each well for a single-step RT-qPCR on a 7500 Fast Real-Time PCR System (Applied Biosystems) in standard mode. Cycling consisted of one cycle of 45°C for 10 min for the reverse transcription of RNA to cDNA, followed by one cycle at 95°C for 10 min. The cDNA was amplified by PCR for 45 cycles, each cycle consisting of 95°C for 15 s and 60°C for 45 s. A negative result or not detected, was determined if no amplification occurred (i.e. the C_T_ value was greater than or equal to 40 cycles) as per Smith *et al*. (2001) [17].

### Immunohistochemistry

All PCR-positive animals for which paraffin wax blocks were available were tested by immunohistochemistry (IHC). Sections were cut onto glass slides and dewaxed and hydrated in xylene and graded ethanol to water. Antigens were visualised using an immunohistochemistry procedure, as follows. To retrieve antigens, sections were heated in a commercial buffer (Envision Flex target retrieval solution High pH, Dako) for 20 min at 97°C, then the sections were rinsed in TRIS buffer (pH 7.6). Endogenous peroxidase was blocked with 3% aqueous H_2_O_2_ for 10 min; the primary antibody was added for 45 min, followed by the secondary antibody conjugate (Envision Flex/HRP, Dako) for 20 min; each step was followed by rinsing in TRIS buffer. Then 3-amino-9-ethylcarbazole (AEC) was added for 10 min to develop colour, followed by a distilled water rinse. The sections were counterstained with haematoxylin. The primary antibody was a rabbit antiserum raised against purified recombinant-expressed Nipah virus nucleoprotein, as described previously [18].

### Statistical Analysis

Test results from RT-qPCR (n = 310) were subjected to a generalised linear model (GLM) [19] under the Binomial distribution and logit link, using GenStat [20]. Five descriptive variables were available for analysis by GLM: reproductive status, season, species, location and year. Reproductive status was similar to that used by Plowright *et al* [14]: male, immature female, mature female not pregnant, mature female pregnant and unknown. Seasons in the southern hemisphere are spring (Sep-Nov), summer (Dec-Feb), autumn (Mar-May) and winter (Jun-Aug). Location denotes the bat submission location north or south of the Tropic of Capricorn (23°26’S), which divides Queensland into the southern temperate zone and the northern tropical zone [21].

## Results

### Reverse Transcription Quantitative Polymerase Chain Reaction

This study screened a total of 673 tissues from 310 animals. Of the 295 flying-foxes tested, 20 individuals returned a positive result (Table 1) with C_T_-values ranging from 26 to 39 (Table 2). Detections were most prevalent in *P*. *conspicillatus* and *P*. *alecto*, with 10 (22.7%) and 9 (8.6%) respectively. There was a single detection in *P*. *poliocephalus* (1%), and no detections in *P*. *scapulatus* (n = 50), unknown *Pteropus* (n = 5) or the Vespertilioniformes (n = 15). From the 20 HeV positive flying-foxes, a total of 30 tissues tested positive, predominantly spleen, which yielded 18 (94.7%) detections, and kidney, which yielded 5 (31.3%) detections. Liver, lung, red blood cells, serum and placenta also yielded positive results (Table 2). All foetal tissues from HeV positive (n = 4) and negative (n = 25) pregnant females tested negative. HeV was detected in the placenta of one pregnant *P*. *alecto* (Bat 9, Table 2), but there was no evidence of HeV RNA in the foetal spleen, liver or lung (Bat 9–1, Table 2). HeV was not detected in placental tissues from the remaining HeV positive (n = 2) and negative (n = 24) pregnant females for which placental tissues were available (n = 27).

**Table 2:** Description and results for 20 Hendra virus positive flying-foxes tested by RT-qPCR.

Bat	Species	Sex	Age	Reproductive status	Tissue^a^ (Ct)^b^									
					Sp	Ki	Li	Lu	Pl	Or	Pr	Ur	RBC	Se
1	*P*. *conspicillatus*	Unknown	Unknown	Unknown	33	ND	ND	ND						
2	*P*. *conspicillatus*	Male	Immature	Male	37	35	ND	ND						ND
3	*P*. *conspicillatus*	Male	Mature	Male	36	ND	ND	ND						
4	*P*. *conspicillatus*	Female	Mature	Mature Female Pregnant	ND	37	ND	ND						
4–1	*P*. *conspicillatus*	Unknown	Foetus	N/A			ND	ND						
5	*P*. *conspicillatus*	Female	Mature	Mature Female Not Pregnant	32	35	ND	ND						
6	*P*. *alecto*	Male	Mature	Male		38	ND	ND						
7	*P*. *alecto*	Female	Mature	Mature Female Pregnant	36	ND	ND		ND			ND	ND	
7–1	*P*. *alecto*	Unknown	Foetus	N/A			ND							ND
8	*P*. *alecto*	Female	Mature	Mature Female Pregnant	36	ND	ND	ND	ND					ND
8–1	*P*. *alecto*	Unknown	Foetus	N/A		ND	ND	ND						
9	*P*. *alecto*	Female	Mature	Mature Female Pregnant	26	36	36	35	31					ND
9–1	*P*. *alecto*	Unknown	Foetus	N/A	ND		ND	ND						
10	*P*. *alecto*	Female	Mature	Mature Female Not Pregnant	32	ND	ND	ND						ND
11	*P*. *alecto*	Female	Mature	Mature Female Not Pregnant	37	ND	ND	ND		ND			ND	
12	*P*. *conspicillatus*	Unknown	Unknown	Unknown	34	ND	ND	ND						
13	*P*. *alecto*	Female	Mature	Mature Female Not Pregnant	36		ND	ND						
14	*P*. *conspicillatus*	Male	Mature	Male	38		ND	ND						ND
15	*P*. *conspicillatus*	Female	Mature	Mature Female Not Pregnant	34		ND	ND						ND
16	*P*. *conspicillatus*	Female	Mature	Mature Female Not Pregnant	28		34	ND					31	35
17	*P*. *conspicillatus*	Male	Immature	Male	35	ND		ND						
18	*P*. *poliocephalus*	Male	Mature	Male	32	ND	ND	37			ND		ND	ND
19	*P*. *alecto*	Female	Mature	Mature Female Not Pregnant	36	ND	ND	ND						
20	*P*. *alecto*	Female	Mature	Mature Female Not Pregnant	39	ND	ND	ND						

^a^ Spleen (Sp), kidney (Ki), liver (Li), lung (Lu), placenta (Pl), oral swab (Or), preputial swab (Pr), urine (Ur) red blood cells (RBC) and serum (Se) not analysed (N/A). The absence of results on brain reflects the non-availability of this tissue for these individuals. Brain tissue samples from most individuals in the archive had been utilised in an earlier study screening for Australian bat lyssavirus infection.

^b^ A positive result, or detection of Hendra virus in available tissue, was recorded when amplification occurred and the reported C_T_ value was less than 40 cycles. Otherwise the available tissue was recorded as not detecting Hendra virus (ND).

### Immunohistochemistry

Of the 20 individuals positive by PCR, 18 had paraffin wax blocks available, including lung, spleen and lymph nodes, liver, salivary gland, kidney, muscle and gonads. Henipavirus antigen was not detected in any tissue.

### Statistical analysis

Analysis by GLM indicated that the adjusted mean prevalence of HeV in the dataset was 6.4%. Species was the only significant variable (p = 0.003); reproductive status, season, year and location were not significant (p > 0.05). Adjusted mean proportions and their standard errors are reported (Table 1).

## Discussion

This archived tissue collection has presented a unique opportunity to examine both HeV tissue tropism and risk factors for infection in naturally infected flying-foxes of the four Australian species of the genus *Pteropus*. HeV was detected in multiple tissues including kidney, spleen, lung, liver, placenta and blood components, consistent with previous studies of *P*. *alecto* and *P*. *poliocephalus* [9–11]. However, this study represents the first detection of HeV RNA in the tissues of *P*. *conspicillatus*, with detections in spleen, kidney, liver and blood components.

The apparent predilection of HeV for the spleen in naturally-infected flying-foxes is consistent with previous experimental infection studies [11]. These combined findings suggest that the spleen may play a fundamental role in either the maintenance of infection or the processing of viral components in the infected flying-fox. Firstly, detection of HeV RNA in the spleen could indicate that this organ is an active site of viral replication, as live virus has been isolated from respective foetal and adult spleens in two of four experimentally-infected pregnant grey-headed flying-foxes [10]. While IHC staining in the vascular tissue of the spleen was an inconsistent finding in experimentally-infected male *P*. *poliocephalus* (two of eight animals) [9], it nonetheless also supports the possibility that the spleen could be an active site of virus replication in infected flying-foxes. Secondly, detection of HeV in the spleen could simply reflect the processing of viral components in this immunologically-active tissue. A third possibility is that the spleen represents a site of latent infection in flying-foxes, and a plausible mechanism for the hypothesized role of recrudescence in the persistence of HeV infection in flying-fox populations [22,23]. It is not possible to differentiate between these hypotheses on the basis of PCR testing alone, and while our negative IHC findings in this study constrain further interpretation, we suggest that the alternative scenarios are not necessarily mutually exclusive. Regardless of whether virus replicates in the spleen or at a distant site, circulating via blood and lymph to sequester and concentrate in the spleen, it is evident that the spleen is the tissue of choice in determining the infection status of an individual. Future immunological studies exploring the structure and function of bat follicular dendritic cells (FDCs) may clarify the role of the spleen.

While viral RNA was detected in tissues, no antigen was detected in corresponding formalin-fixed paraffin embedded tissue sections by IHC. This finding is consistent with that of Halpin *et al*. (2011), who also reported negative IHC results in PCR-positive tissue samples from experimentally-infected *P*. *alecto* (including kidney and bladder samples from a single non-pregnant female bat in which HeV was isolated in urine 12–14 days prior to euthanasia). Thus, the negative IHC results in our study likely reflect the lower diagnostic sensitivity of that assay compared to PCR.

While the exact mode of HeV transmission in flying-foxes is unknown, the detection of HeV RNA in the spleen, liver and lung of naturally-infected flying-foxes demonstrates that systemic infection almost certainly occurs, presumably following local replication in the exposed mucosal surfaces. It is possible that HeV could progressively track from upper (e.g. nasal mucosa) to lower (e.g. lung) respiratory tract tissues without systemic involvement, and the portal vein draining the gastrointestinal tract could theoretically translocate HeV from the gut to the liver without precipitating a truly systemic infection, presuming an oral route of exposure. However it is improbable that detection of HeV in the spleen could be explained through such direct transmission pathways. The detection of HeV RNA in kidney tissue also reflects likely initial mucosal replication at the portal of entry (e.g. oronasal) followed by systemic infection and localization in the kidneys. Though again improbable, the possibility of a retrograde-type urinary tract infection in naturally infected flying-foxes (whereby viral replication in lower urogenital tissues precedes viral spread via urethral, bladder, ureteral and finally, kidney tissues cannot be totally excluded). Local replication at the site of inoculation with subsequent systemic infection has been demonstrated in several experimental HeV and NiV infection trials in *Pteropus* spp. [9–11,18]. Our findings in naturally infected flying-foxes support this basic model of infection for henipaviruses in their reservoir hosts.

Vertical transmission of HeV has been demonstrated previously in experimentally infected [10] and naturally infected [2,10] flying-foxes. The absence of HeV detection in foetal tissue in all four HeV-positive pregnant animals in this study indicates that *in-utero* transmission does not always occur. The finding has relevance to both natural HeV infection dynamics in flying-foxes and to the hypothesized role of infected placental and foetal tissues as a source of infection in equine spillovers [2,10]. While limited numbers preclude any meaningful estimate of frequency of vertical transmission, the lack of detections in foetal tissues in our study supports previous contentions (based on seroprevalence patterns in three species) that it is unlikely to be the predominant form of transmission [1,13,14]. In these studies, the low seroprevalence observed in juvenile and immature flying-foxes after maternally-acquired passive immunity had waned was not considered to be consistent with a high frequency of HeV exposure *in utero*.

The first two detected HeV spillovers to horses were temporally correlated with the southern hemisphere spring birthing season of three of the four Australian mainland species of *Pteropus*, leading to postulation of flying-fox birthing as a risk factor for spillover [24]. However, as spillover numbers have accumulated over intervening years, a strong temporal clustering of spillovers during the southern hemisphere winter is increasingly evident [12], now with 35/51 spillovers to 30 June 2014 occurring in June, July and August. This temporal association coincides with mid- to late-gestation in *P*. *alecto*, *P*. *conspicillatus* and *P*. *poliocephalus* rather than the birthing season *per se*, and while unseasonal births do occur throughout the year, the main birthing pulse in these species occurs in late September to November [25]. Indeed, the described reproductive biology of flying-foxes also argues against infected placental and foetal tissues as a likely source of infection in equine spillovers: Hall & Richards (2000) observed that flying-foxes have diurnal parturition that takes place in the roost with the mother consuming the expelled placenta [25].

When investigating effective risk management strategies for disease spillover from flying-foxes to horses and humans, it is important to understand infection dynamics in the host species. Analysis indicates that flying-fox species is a risk factor for HeV infection, with the two closely related species *P*. *alecto* and *P*. *conspicillatus* [26] evidently the primary reservoir host of HeV. Notwithstanding the modest sample size in our study, this contention is supported by the absence of HeV detection in *P*. *scapulatus* and the detection of HeV in only one *P*. *poliocephalus*. These differential species detections provide biological support for a recent modelling study that showed a positive spatial correlation between the combined density of *P*. *alecto* and *P*. *conspicillatus*, and the location of equine spillovers [16]. Taken together, the two studies suggest that *P*. *alecto* and *P*. *conspicillatus* play a particularly important role in the cross-species transmission of HeV infection to horses.

The lack of HeV detection in *P*. *scapulatus* tissues in this study (95% CI 0–7%) is consistent with the findings of Field *et al*. (2011) [12] and Edson *et al*. (in preparation), and suggests that systemic infection is infrequent or lacking in this species, however, all four mainland Australian species of *Pteropus*, including *P*. *scapulatus*, have antibodies for HeV [1,13,14]. We suggest that exposure to HeV in *P*. *scapulatus* may result in seroconversion in the absence of systemic replication and/or shedding of virus. However, the possibility of local replication and/or shedding cannot be excluded, given the recent description in a mouse model of HeV infection, characterized by the absence of viremia or systemic involvement [27]. If *P*. *scapulatus* are able to effectively clear HeV infection before systemic involvement, it is possible that HeV RNA could be present in tissues (e.g. oral cavity) or secretions (e.g. oronasal secretions) that were unavailable for testing in this study. However, if our contention that *P*. *scapulatus* seroconverts in the absence of systemic replication and virus excretion is correct, this would suggest that serology may not be a robust proxy for infection and transmission data when viral excretion has not been demonstrated.

The role of *P*. *poliocephalus* in HeV infection dynamics is less clear. While there is demonstrated HeV detection in this species in this and previous studies through detection of RNA, virus isolation, and IHC [2,9,10], the significantly lower infection prevalence found in this study (1%: 95% CI 0.03–7%) suggests *P*. *poliocephalus* has limited viral replication and/or excretion. This finding is again supported by the modelling of Smith *et al*.(2014) [16], and may have implications for experimental HeV infection studies, the majority of which have used *P*. *poliocephalus* [9,10], and it may be that *P*. *alecto* and *P*. *conspicillatus* are better model species.

Our analysis identified species as a significant risk factor for HeV infection, but not reproductive status, season, latitude and year. Seroprevalence studies have previously reported risk factors for HeV as age, pregnancy and lactation in *P*. *conspicillatus* [13], and age, sex, pregnancy, lactation, and season in *P*. *scapulatus* [14]. Whether this lack of agreement reflects sample size limitations in our study, or the different outcome variables used (HeV detection in this study verses HeV sero-status in the earlier studies) is unclear at this time.

## Conclusion

Our study has yielded a number of significant findings including the first detection of HeV in the tissues of *P*. *conspicillatus*, providing evidence of systemic infection in this species. The predilection of HeV for the spleen of naturally infected flying-foxes suggests that the spleen may play a vital role in either the maintenance of infection, immunological processing of viral components or a mechanism for recrudescence. Our lack of detection of infection in foetal tissues of HeV positive pregnant females supports placental and foetal tissues not being an important source of equine infection, and reinforces flying-fox urine as a key transmission pathway. Finally, we have shown that species is a risk factor for the detection of HeV in naturally infected flying-foxes, specifically *P*. *alecto* and *P*. *conspicillatus*, supporting the contention of Smith *et al*. (2014) that these two species are likely the primary reservoir host of HeV, and play a major role in the cross-species transmission of HeV infection to horses.

Additional research that investigated routes of excretion in naturally infected flying-foxes, and thus the modes of transmission to horses would complement the findings of this study, and enable better targeted HeV exposure risk management advice to the horse industry.
